# Regional Validation of Coronary Heart Disease Risk Factors in Psoriasis: A Multicenter Study in Southern China

**DOI:** 10.3390/jcm15218384

**Published:** 2026-10-28

**Authors:** Yuying Yao, Martin Man Ho Chung, Zhengfeng Li, Yu Lan, Shaozhen Mai, Liuyu Chen, Xiaoyan Wu, Yu Zhang, Christina Sze Man Wong, Zhenying Zhang

**Affiliations:** 1 Department of Dermatology, The Eighth Affiliated Hospital, Sun Yat-sen University, Shenzhen 518033, China; yaoyy56@mail.sysu.edu.cn (Y.Y.); lany29@mail.sysu.edu.cn (Y.L.); maishzh5@mail.sysu.edu.cn (S.M.); chenly96@mail.sysu.edu.cn (L.C.); 2 Division of Dermatology, Department of Medicine, Li Ka Shing Faculty of Medicine, The University of Hong Kong, Hong Kong, China; drcmhm@hku.hk; 3 Division of Dermatology, Department of Medicine, Queen Mary Hospital, Hong Kong, China; 4 Department of Dermatology, The University of Hong Kong-Shenzhen Hospital, Shenzhen 518053, China; lizf@hku-szh.org (Z.L.); wuxy7@hku-szh.org (X.W.); zhangy1@hku-szh.org (Y.Z.)

**Keywords:** psoriasis, comorbidities, coronary heart disease, risk factors

## Abstract

**Background:** Psoriasis is a systemic inflammatory disorder associated with cardiovascular comorbidities. Although an association between psoriasis and coronary heart disease (CHD) has been established, data from southern Chinese populations remain limited. **Objective**: The purpose was to examine the factors associated with CHD in patients with psoriasis in a southern Chinese population. **Methods**: We conducted a multicenter, retrospective case–control study across three hospitals. This study included 56 cases (psoriasis with CHD) and 112 age-matched controls (psoriasis without CHD). The data were obtained from a pre-established psoriasis-specific database (September 2023–February 2025). Univariate analysis used binary logistic regression; multivariable conditional logistic regression accounted for the 1:2 age-matched design, with sex and smoking forced into the model. **Results:** In the multivariable conditional logistic regression analysis, greater body surface area (BSA) involvement (OR = 1.034, 95% CI: 1.009–1.059, *p* = 0.007), diabetes (OR = 4.748, 95% CI: 1.490–15.132, *p* = 0.008), and dyslipidemia (OR = 33.869, 95% CI: 4.549–252.136, *p* < 0.001) were significantly associated with CHD. Hypertension and smoking showed positive but non-significant associations. **Conclusions:** Greater BSA involvement, diabetes, and dyslipidemia were associated with CHD in this southern Chinese psoriasis cohort, consistent with previous international cohorts. These associations should be interpreted as cross-sectional rather than causal.

## 1. Introduction

Psoriasis is a common, chronic, and relapsing autoimmune inflammatory disorder, clinically characterized by erythematous plaques covered with silvery scales [1]. It affects 2–4% of the population in Western countries [2]. Although the reported prevalence in China is lower (approximately 0.47%), the sheer size of the Chinese population places the country among those with the largest absolute number of psoriasis patients worldwide [3].

Over the past decade, psoriasis has increasingly been recognized as a systemic disease rather than a purely dermatological condition. Beyond the skin, it is often accompanied by comorbidities involving the cardiovascular, endocrine–metabolic, and gastrointestinal systems. Such comorbid conditions not only exacerbate the severity and progression of psoriasis but also complicate treatment strategies, frequently leading to suboptimal outcomes and diminished quality of life [4]. Among these comorbidities, cardiovascular disease—particularly coronary heart disease (CHD)—has drawn significant attention due to its high rates of disability and mortality in psoriasis cohorts [5].

An association between psoriasis and CHD has been well documented [6,7,8,9,10]. Moreover, diabetes, dyslipidemia, and extensive body surface area (BSA) involvement have already been established as CHD risk factors in psoriasis across multiple large international cohorts. However, data from Asian populations—particularly southern Chinese patients—remain limited, and whether these established risk factors show the same patterns in this regional context has not been fully elucidated. From a preventive medicine perspective, validating these associations in diverse populations is essential for developing targeted interventions to reduce CHD incidence in this vulnerable group. To address this regional evidence gap, we conducted a retrospective case–control study comparing Chinese psoriasis patients with and without CHD, aiming to confirm established risk factors and explore additional demographic and clinical characteristics associated with CHD in this population.

## 2. Methods

### 2.1. Study Design

We conducted a multicenter, hospital-based, retrospective case–control study across three participating institutions: the University of Hong Kong-Shenzhen Hospital, the Eighth Affiliated Hospital of Sun Yat-Sen University, and Queen Mary Hospital. The study was carried out in compliance with the Declaration of Helsinki and received ethical approval from the institutional review board of each participating hospital (Institutional Review Board of The Eighth Affiliated Hospital, Sun Yat-sen University (approval no. 2025-075-01; approval date 23 June 2025); Institutional Review Board of The University of Hong Kong-Shenzhen Hospital (approval no. [2025] 169; approval date 20 June 2025); Institutional Review Board of The University of Hong Kong/Hospital Authority Hong Kong West Cluster (approval no. UW 25-155; approval date 31 March 2025)).

### 2.2. Participants

We identified and screened psoriasis patients from the participating hospitals between September 2023 and February 2025 using our institutional database. Case patients were defined as individuals of any age diagnosed with both psoriasis and CHD, with documented psoriasis diagnosis preceding that of CHD. Psoriasis diagnoses were based on the following ICD-10 codes: L40.000, L40.001, L40.102, L40.400, L40.500, L40.802, and L40.900. CHD included ischemic heart disease, myocardial infarction (acute (I21), subacute (I22), or chronic (I25.1, I25.2)), and angina pectoris (I20). Control participants consisted of psoriasis patients without any recorded history of CHD, defined as having at least one psoriasis-related ICD-10 code and no CHD diagnostic records.

To enhance comparability and minimize confounding, each case was matched with two controls based on age (±2 years) using propensity score matching performed with the MatchIt package in R version 4.4.2. Matching was conducted via the nearest neighbor method with a 1:2 ratio and a caliper width of 0.3. Sex and smoking were not used for matching but were forced into the multivariable conditional logistic regression model. Patients with missing or incomplete clinical data were excluded from the analysis to ensure data integrity and reliability.

We defined an index date for all participants to provide a comparable reference point. For cases, the index date was the date of CHD diagnosis. For each control, the index date was the CHD diagnosis date of the matched case. The case definition required that psoriasis was diagnosed before CHD; however, no temporal requirement could be applied to the other variables because their diagnosis or assessment dates were not reliably recorded in the source database.

### 2.3. Procedures

Data for this retrospective analysis were extracted from a pre-established psoriasis-specific database, which was built upon electronic medical record systems from three hospitals. Extraction followed international classification of diseases, 10th revision (ICD-10) coding standards. The database documented clinical information of patients seen between September 2023 and February 2025. Retrieved variables included gender, age, age at onset, disease duration, body surface area (BSA) score, lesion distribution (scalp, genitals, nails), psoriasis subtype (vulgaris, erythrodermic, pustular, psoriatic arthritis), and comorbidities (CHD, obesity, hypertension, diabetes, and dyslipidemia).

Body mass index (BMI) was calculated as weight (kg)/height squared (m^2^). According to the Chinese Guidelines for the Diagnosis and Treatment of Obesity (2024 edition), obesity is defined as having a BMI of 28 kg/m^2^ or higher among the adult population in China. The diagnosis of hypertension, diabetes and dyslipidemia is determined by specialized physicians. We extracted relevant data information from the medical record system through diagnostic codes. Each comorbidity was considered present only if it had been explicitly documented as a confirmed diagnosis in the medical records, which we retrieved based on ICD-10 codes. Specifically,

- Hypertension was identified using ICD-10 codes I10, I11, I12, I13, I14, and I15.
- Diabetes was identified using ICD-10 codes E10–E14 (including E10, E11, E12, E13, and E14).
- Dyslipidemia was identified using ICD-10 codes E78.

Importantly, diagnosis dates for BSA, obesity, hypertension, diabetes, dyslipidemia, smoking, and biologic exposure were not reliably recorded in the source database; therefore, these variables were treated as concurrent conditions, and no temporal precedence relative to CHD could be established.

The types of biological agents used were also systematically recorded, as shown below. Biologics switching was defined as the use of biologics targeting two or more distinct targets.

- TNF inhibitors: adalimumab and etanercept;
- Interleukin-17 (IL-17) inhibitors: secukinumab and ixekizumab;
- IL-23 inhibitors: guselkumab and risankizumab;
- IL-12/23 inhibitor: ustekinumab.

### 2.4. Statistical Analysis

Continuous variables are presented as median (interquartile range, IQR) and categorical variables as numbers (percentages). In univariate analysis, each variable was entered into a separate binary logistic regression model to assess its association with CHD. Variables with a *p*-value < 0.10 in univariate analysis, together with sex and smoking status, were included in the multivariable model. Sex and smoking were forced into the model regardless of their univariate significance because they are major confounders for CHD.

Since cases and controls were matched 1:2 by age, the multivariable analysis used conditional logistic regression to account for the matched design. Conditional logistic regression was implemented using the Cox regression procedure in SPSS, with the matched set identifier as the stratification variable and a virtual time variable (2 − CHD) to define event status. This approach is equivalent to conditional logistic regression for matched case–control data. Results are expressed as odds ratios (OR) with 95% confidence intervals (CIs). A two-sided *p*-value < 0.05 was considered statistically significant. All analyses were performed using SPSS version 27 (IBM, SPSS Statistics 27).

Missing data and selection bias were treated as follows. The primary analysis used complete-case data. We compared included (*n* = 521) and excluded (*n* = 593) patients on available baseline characteristics (Supplementary Table S1). As a sensitivity analysis, we applied inverse probability of inclusion weighting (IPW): inclusion probability was modeled using available baseline variables; included patients were weighted by 1/*p*, with weights truncated at the 1st and 99th percentiles, so that the weighted sample represented the original 1114 patients; IPW-weighted logistic regression was then performed. IPW was used only as a sensitivity analysis and did not replace the primary matched conditional logistic regression.

## 3. Results

### 3.1. Cases and Controls

**Figure 1:**
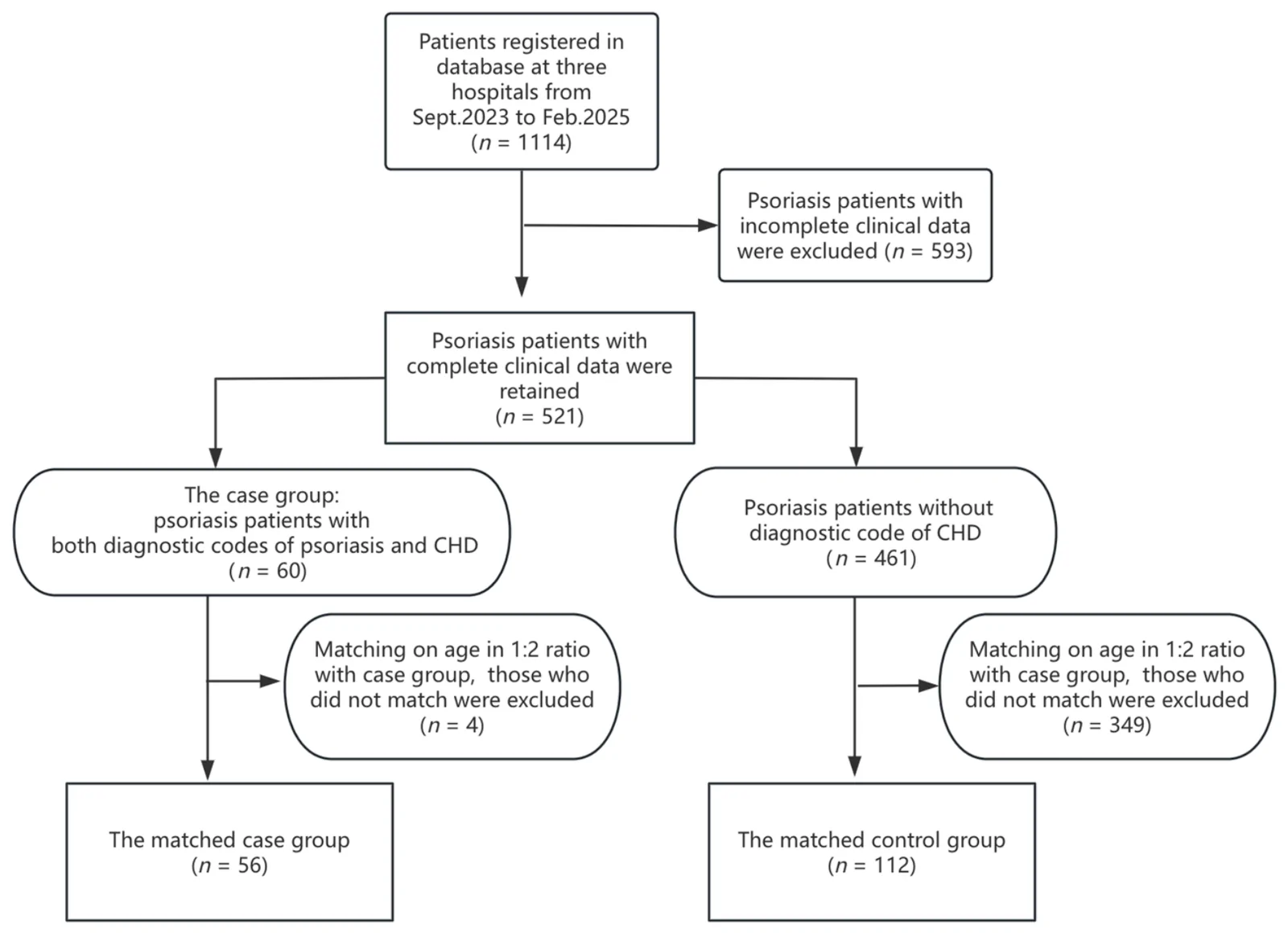
Study flow diagram. CHD: coronary heart disease.

A total of 1114 psoriasis patients from the three hospitals met the inclusion criteria. After excluding 593 patients due to missing clinical data essential for this study, 521 eligible patients remained, comprising 60 in the case group and 461 in the control group. Using propensity score matching, cases and controls were matched at a 1:2 ratio based on age. Ultimately, 56 cases and 112 controls were successfully matched, the remaining 4 cases and 349 controls that could not be matched were excluded from the analysis (Figure 1).

The included and excluded patients had similar available baseline characteristics (all *p* > 0.05; Supplementary Table S1), and the IPW sensitivity analysis showed directionally consistent associations with the primary analysis (Supplementary Tables S2 and S3), suggesting that the main findings were not substantially driven by selection bias, although residual bias cannot be excluded.

### 3.2. Comparison of Demographic Characteristics and Risk Factors Between Cases and Controls (Univariate Analysis)

A univariate analysis comparing demographic characteristics and risk factors between cases and controls is presented in Table 1. In the binary logistic regression analysis, hypertension (64.29% vs. 32.14%), diabetes (44.64% vs. 14.29%), dyslipidemia (50.00% vs. 11.61%), and BSA (median 15% vs. 6.5%) were significantly associated with CHD (all *p* < 0.001). Smoking showed a borderline association (48.21% vs. 33.03%, *p* = 0.058).

No significant associations were observed for sex, age at onset, disease duration, obesity, psoriasis subtype, nail/scalp/genital involvement, biologic treatment, or duration of biologics (all *p* > 0.05). Variables with *p* < 0.10 in univariate analysis (smoking, hypertension, diabetes, dyslipidemia, and BSA), together with sex, were included in the multivariable conditional logistic regression model; sex was forced into the model irrespective of its univariate *p* value.

### 3.3. Risk Factors Associated with CHD Occurrence in Psoriasis (Multivariate Analysis)

In the multivariable conditional logistic regression analysis, which accounted for the 1:2 age-matched design and included variables identified in univariate analysis (BSA, hypertension, diabetes, and dyslipidemia) together with sex and smoking. BSA (OR = 1.034, 95% CI: 1.009–1.059, *p* = 0.007), diabetes (OR = 4.748, 95% CI: 1.490–15.132, *p* = 0.008), and dyslipidemia (OR = 33.869, 95% CI: 4.549–252.136, *p* < 0.001) were significantly associated with CHD occurrence in this hospital-based psoriasis population (Table 2). Hypertension (OR = 2.502, 95% CI: 0.808–7.751, *p* = 0.112) and smoking (OR = 2.781, 95% CI: 0.873–8.857, *p* = 0.083) showed positive associations but did not reach statistical significance. Gender (OR = 1.173, 95% CI: 0.278–4.958, *p* = 0.828) was not significantly associated with CHD.

## 4. Discussion

In this multicenter, retrospective, hospital-based case–control study across three Chinese hospitals, we analyzed psoriasis patients recorded between September 2023 and February 2025. We aimed to examine the factors associated with CHD in a southern Chinese psoriasis population and to assess whether established risk factors show similar patterns in this regional context. Greater BSA involvement and concurrent dyslipidemia or diabetes were associated with CHD, consistent with previous international cohorts.

Since exposure dates were unavailable, we could not determine whether BSA, metabolic comorbidities, smoking, or biologic exposure preceded CHD. Reverse causation and surveillance bias are possible. The observed associations should be interpreted as cross-sectional rather than causal.

BSA reflects not only the extent of cutaneous involvement but also the systemic inflammatory burden. PET/CT studies have shown increased aortic inflammation in psoriasis that correlates with disease severity [11]. Chronic skin inflammation contributes to insulin resistance and endothelial dysfunction, promoting metabolic syndrome and atherogenesis [12]. Consistent with prior reports, cases had a higher mean BSA than controls, supporting the generalizability of the BSA–CHD association to southern Chinese patients.

Psoriasis is linked to multiple metabolic syndrome components, including diabetes, insulin resistance, dyslipidemia, hypertension, and central adiposity [13]. In our study, concurrent dyslipidemia and diabetes were associated with CHD. These findings align with evidence linking psoriasis to metabolic comorbidities [14,15,16,17] and extend this evidence to a Chinese cohort. However, diagnosis dates for diabetes and dyslipidemia were not reliably recorded; therefore, we could not determine whether they preceded CHD. Reverse causation and surveillance bias are possible, because a CHD diagnosis may prompt more frequent glucose and lipid testing. These associations should be interpreted as cross-sectional rather than causal.

Psoriasis is characterized by Th1- and Th17-derived cytokines (e.g., IL-17, IL-23, TNF-α) that modulate angiogenesis, insulin signaling, adipogenesis, and lipid metabolism and have been implicated in obesity, insulin resistance, thrombosis, and atherosclerosis. Conversely, inflammatory mediators from metabolic syndrome and atherosclerotic lesions may exacerbate psoriatic inflammation, potentially creating a self-perpetuating cycle [18,19]. Shared genetic susceptibility and overlapping cytokine pathways further link immune-mediated inflammatory diseases with metabolic and cardiovascular comorbidities [18]. Understanding these relationships is essential for improving outcomes in diverse populations.

Although biologics targeting these cytokines may mitigate cutaneous and systemic inflammation [20], we found no significant difference in their use between cases and controls. The retrospective design and short duration of biologic administration limited the assessment of their impact; larger prospective studies with longer follow-up times are needed.

In summary, in this southern Chinese cohort, greater BSA involvement and concurrent dyslipidemia or diabetes were associated with CHD in psoriasis patients, confirming the findings from previous international cohorts and extending this evidence to a southern Chinese population. Since diagnosis dates for these metabolic comorbidities were unavailable, the associations should be interpreted as cross-sectional rather than causal. Patients with severe skin disease or metabolic abnormalities may warrant regular cardiovascular screening. The limitations include the small tertiary-center sample, the reliance on ICD-10 coding, and the inability to establish temporal sequence, which may introduce residual reverse causation and surveillance bias. Moreover, the high exclusion proportion (53.2%) remains a limitation; IPW can only adjust for observed variables, and residual selection bias cannot be fully excluded. Prospective studies with standardized timing are needed to confirm these findings.

## Figures and Tables

**Table 1:** Demographic characteristics and risk factors in case and matched control patients (results of univariate analysis).

	Case (*n* = 56)	Control (*n* = 112)	*p*
Gender			
Male	45 (80.36%)	84 (75%)	0.439
Female	11 (19.64%)	28 (25%)	
Smoking	27 (48.21%)	37 (33.03%)	0.058
Age of onset (year)	45.5 (34.25–62)	48 (39–61)	0.528
Duration of psoriasis (year)	14 (5–23)	10 (5–20)	0.325
Obesity	7 (12.5%)	12 (10.71%)	0.731
Hypertension	36 (64.29%)	36 (32.14%)	<0.001
Diabetes	25 (44.64%)	16 (14.29%)	<0.001
Dyslipidemia	28 (50%)	13 (11.61%)	<0.001
Subtype of psoriasis			0.148
Psoriasis vulgaris	43 (76.79%)	95 (84.82%)	
Arthritic psoriasis	9 (16.07%)	16 (14.29%)	
Erythrodemic and pustular psoriasis	4 (7.14%)	1 (0.89%)	
BSA (%)	15 (5.25–48.75)	6.5 (3–14.25)	<0.001
Presence of nail psoriasis	20 (35.71%)	34 (30.36%)	0.484
Scalp involvement	32 (57.14%)	63 (56.25%)	0.912
Genital involvement	5 (8.93%)	15 (13.39%)	0.403
Treatment of biologics	30 (53.57%)	66 (58.93%)	0.509
TNF inhibitor	1	2	
IL-17 inhibitor	19	38	
IL-23 inhibitor	6	22	
IL12/23 inhibitor	1	1	
Biologics switching	3	3	
The duration of biologics			0.607
<1 year	10	17	
≥1 years	20	49	

**Table 2:** Association of the occurrence of CHD with potential risk factors for psoriasis (results of multivariate analysis).

	*p*	OR (95% CI)
Gender	0.828	1.173 (0.278, 4.958)
Smoking	0.083	2.781 (0.873, 8.857)
BSA (%)	0.007	1.034 (1.009,1.059)
Hypertension	0.112	2.502 (0.808, 7.751)
Diabetes	0.008	4.748 (1.490, 15.132)
Dyslipidemia	<0.001	33.869 (4.549, 252.136)

OR: Odds ratio, CI: confidence interval.

## Data Availability

The data that support the findings of this study are available from the corresponding author upon reasonable request.

## Supplementary Materials

The following supporting information can be downloaded at: https://www.mdpi.com/article/10.3390/jcm15218384/s1, Table S1: Comparison of included (*N* = 521) and excluded (*N* = 593) patients; Table S2: IPW-weighted sensitivity analysis of factors associated with CHD (*n* = 521, weighted to represent 1114 patients); Table S3: Consistency between the primary matched conditional logistic regression (*n* = 168) and the IPW-weighted analysis (*n* = 521).
