# Supplementary material for: Regional Validation of Coronary Heart Disease Risk Factors in Psoriasis: A Multicenter Study in Southern China
Source: J Clin Med. 2026 Oct 28;15(21):8384. doi: 10.3390/jcm15218384 (PMC13763689; doi:10.3390/jcm15218384)
Supplement: Supplementary file 1 [file jcm-15-08384-s001.zip › jcm-4577373-supplementary.pdf]

Supplementary

Supplementary Table S1. Comparison of included (*N* = 521) and excluded (*N* = 593) patients. No significant differences were observed in age, gender, smoking, CHD status, hypertension, diabetes, dyslipidemia, obesity, BSA, or other clinical variables (all $p > 0.05$). Missingness in the excluded group is reported.

	**Included (*N* = 521)** *n*/*N* **(%)**	**Excluded (*N* = 593)** *n*/*N* **(%)**	***p***
Gender	0	0	0.317
Age	0	0	0.142
Age of onset（year)	0	82/593 (13.83%)	0.586
Duration of Psoriasis (year)	0	82/593 (13.83%)	0.17
Smoking	0	183/593 (30.86%)	0.868
CHD	0	0	0.51
Obesity	0	216/593 (36.42%)	0.58
Hypertension	0	0	0.849
Diabetes	0	0	0.282
Dyslipidemia	0	0	0.355
Subtype of psoriasis	0	0	0.540
BSA（%)	0	208/593 (35.08%)	0.895
Presence of nail psoriasis	0	64/593（10.79%）	0.948
Scalp involvement	0	81/593（13.66%）	0.903
Genital involvement	0	79/593（13.32%）	0.916
Treatment of biologics	0	78/593（13.15%）	0.359

n represents the number of samples with missing data

Supplementary Table S2. IPW-weighted sensitivity analysis of factors associated with CHD (*n* = 521, weighted to represent 1,114 patients). Hypertension: OR = 2.311 (95% CI 1.090–4.899), $p = 0.029$; diabetes: OR = 2.990 (1.385–6.453), $p = 0.005$;dyslipidemia: OR = 4.637 (2.198–9.779), $p < 0.001$; BSA: OR = 1.016 (1.003–1.030), $p = 0.018$; sex and smoking were not significant.

	p	**OR (95% CI)**
Gender	0.246	1.618 （0.718，3.647）
Smoking	0.306	1.433（0.719，2.853）
Hypertension	0.029	2.311（1.090，4.899）
Diabetes	0.005	2.990（1.385，6.453）
Dyslipidemia	<0.001	4.637 (2.198, 9.779）
BSA（%）	0.018	1.016 (1.003, 1.030)

Supplementary Table S3. Consistency between the primary matched conditional logistic regression (n = 168) and the IPW-weighted analysis (n = 521). Effect directions were consistent for all main variables; BSA and dyslipidemia were significant in both analyses; diabetes and hypertension were significant only in the IPW analysis; no variable showed a contradictory direction.

**Variable**	**Primary analysis: matched conditional logistic regression (n = 168)**	**Sensitivity analysis: IPW-weighted full cohort (n = 521)**	Consistency
BSA (%)	OR = 1.035 (1.004–1.066), p = 0.025	OR = 1.016 (1.003–1.030), p = 0.018	Direction consistent; both significant
Dyslipidemia	OR = 33.393 (3.106–359.012), p = 0.004	OR = 4.637 (2.198–9.779), p < 0.001	Direction consistent; significance consistent
Diabetes	OR = 2.417 (0.57–10.24), p = 0.231	OR = 2.990 (1.385–6.453), p = 0.005	Direction consistent; significance inconsistent
Hypertension	OR = 1.282 (0.331–4.971), p = 0.719	OR = 2.311 (1.090–4.899), p = 0.029	Direction consistent; significance inconsistent
Gender	OR = 1.391 (0.278–6.954), p = 0.687	OR = 1.618 (0.718–3.647), p = 0.246	Direction consistent; significance consistent (both not significant)
Smoking	OR = 53747.01 (0–2.018E + 52), p = 0.845	OR = 1.433 (0.719–2.853), p = 0.306	Direction not interpretable in primary analysis; not significant in IPW
